# Laparoscopic deroofing to treat an infected hepatic cyst because of fistula formation between the hepatic cyst and the duodenum ulcer

**DOI:** 10.1093/jscr/rjaf484

**Published:** 2025-07-10

**Authors:** Kiyoshi Saeki, Yuki Hattori, Yuki Miyahara, Hiroshi Matsumoto, Tomoki Nakafusa, Kazuhisa Kaneshiro, Hiroshi Kono, Takaharu Yasui, Hirofumi Yamamoto, Takashi Ueki

**Affiliations:** Department of Surgery, Hamanomachi General Hospital, 3-3-1 Nagahama, Fukuoka 810-8539, Japan; Department of Surgery, Hamanomachi General Hospital, 3-3-1 Nagahama, Fukuoka 810-8539, Japan; Department of Surgery, Hamanomachi General Hospital, 3-3-1 Nagahama, Fukuoka 810-8539, Japan; Department of Surgery, Hamanomachi General Hospital, 3-3-1 Nagahama, Fukuoka 810-8539, Japan; Department of Surgery, Hamanomachi General Hospital, 3-3-1 Nagahama, Fukuoka 810-8539, Japan; Department of Surgery, Hamanomachi General Hospital, 3-3-1 Nagahama, Fukuoka 810-8539, Japan; Department of Surgery, Hamanomachi General Hospital, 3-3-1 Nagahama, Fukuoka 810-8539, Japan; Department of Surgery, Hamanomachi General Hospital, 3-3-1 Nagahama, Fukuoka 810-8539, Japan; Department of Surgery, Hamanomachi General Hospital, 3-3-1 Nagahama, Fukuoka 810-8539, Japan; Department of Surgery, Hamanomachi General Hospital, 3-3-1 Nagahama, Fukuoka 810-8539, Japan

**Keywords:** hepatic cyst infection, duodenum ulcer, laparoscopy

## Abstract

We describe a rare case of laparoscopic deroofing to treat an infected hepatic cyst because of fistula formation between the hepatic cyst and the duodenum ulcer. A 71-year-old female was referred to our hospital for the evaluation of her abdominal pain. The laboratory workup revealed a high inflammatory reaction. Computed tomography (CT) visualized a large hepatic cyst in the left hepatic lobe, causing suspicion of a fistulous tract between the hepatic cyst and duodenum. A hepatic cyst infection was diagnosed, and both antibiotic treatment and percutaneous cyst drainage were performed. Although the acute inflammation improved after these treatments, chronic inflammation continued. We conducted laparoscopic deroofing of the infected cyst. The patient’s post-operative course was uneventful, and CT revealed no recurrence 6 months post-procedure. For patients with non-parasitic hepatic cyst infection, physicians should consider not only conservative antibiotic treatment but also surgical treatment including laparoscopic cyst deroofing.

## Introduction

Non-parasitic hepatic cysts are congenital, benign lesions that occur in up to 5%–10% of the population [1–3]. Although most of these cysts are asymptomatic, cyst infection is a severe complication of liver cystic disease.

We provide the details of a rare case of laparoscopic deroofing that was performed for an infected hepatic cyst associated with fistula formation between the hepatic cyst and the duodenum ulcer.

## Case presentation

**Figure 1:**
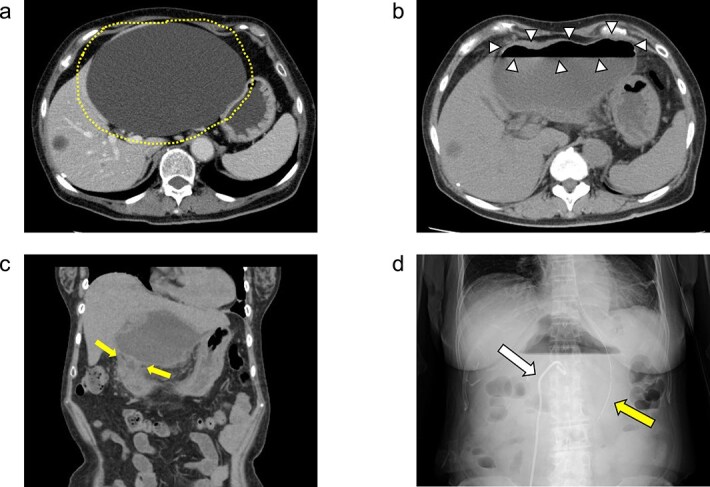
(a) Enhanced CT images findings obtained in the examination by the patient’s primary care physician. A 16-cm hepatic cyst in the left hepatic lobe area was observed. *Dotted line:* The hepatic cyst. (b, c) Plain CT images findings obtained at the patient’s hospitalization. (b) This axial section shows the large hepatic cyst in left hepatic lobe area, which was collapsed and contained air bubbles. There were no findings of free air or leakage into the peritoneal cavity. *Arrowheads:* The air bubbles of collapsed large hepatic cyst. (c) A low-density area between the hepatic cyst and the duodenum with a suspicion of a fistulous tract can be seen on this coronal section. *Arrows:* The low-density area between the hepatic cyst and the duodenum. (d) An abdominal X-ray finding after the percutaneous catheter aspiration of the hepatic cyst with a 7Fr pig-tail catheter (*left arrow*). *Right arrow:* The nasogastric tube.

A 71-year-old female had suffered from upper abdominal pain for ~7–8 years. She consulted her primary care physician, and computed tomography (CT) demonstrated a 16-cm hepatic cyst in the left hepatic lobe (Fig. 1a). Although she had no history of peptic ulcer, she had not gotten an upper gastrointestinal endoscopy for decades.

She exacerbated her upper abdominal pain and was transferred to our hospital. The laboratory workup (Supplementary Figs S1 and S2) showed that her white blood cell count and CRP level were high at 13 300/μl (92.5% neutrophil count) and 41.72 mg/dl, respectively. CT revealed a large hepatic cyst in the left hepatic lobe that was collapsed and contained air bubbles. There were no findings of free air or leakage into the peritoneal cavity (Fig. 1b). The CT also identified a low-density area between the hepatic cyst and the duodenum; a fistulous tract between them was suspected (Fig. 1c).

She was started on antibiotic therapy, and the hepatic cyst was punctured percutaneously with a 7Fr pig-tail catheter (Fig. 1d), and the elevated inflammatory response in blood tests gradually improved.

**Figure 2:**
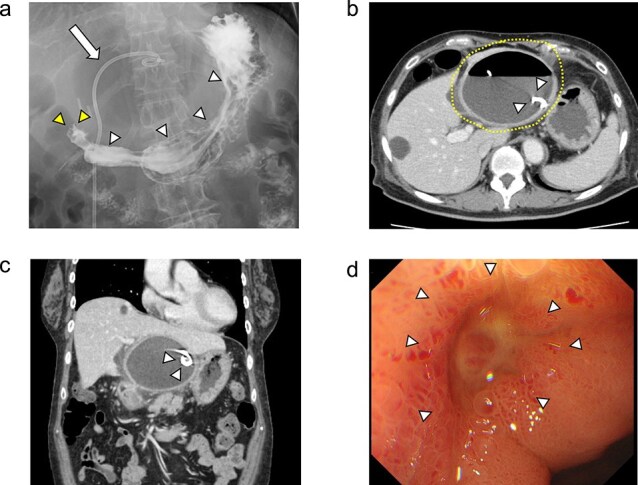
The upper gastrointestinal contrast examination findings on day 13 of the patient’s hospital admission. (a) There was no obvious fistulous tract between the hepatic cyst and the duodenum. *arrowheads:* The stomach, *arrowheads:* The duodenum. *arrow:* The 7Fr pig-tail catheter. (b, c) Enhanced CT images findings on 20 day of admission. (b) Axial section. A shrunken hepatic cyst with a thickened cyst wall was observed. *Dotted line:* The large hepatic cyst. *Arrowheads:* The 7Fr pig-tail catheter. (c) Coronal section. A a low-density area was observed between the hepatic cyst and the duodenum, suspected of being a fistulous tract. *Arrows:* The low-density area between the hepatic cyst and the duodenum. *Arrowheads:* The 7Fr pig-tail catheter. (d) An upper gastrointestinal endoscopy on admission day 21 showed an ulcer scar at the anterior aspect of the duodenum bulb without obvious fistulous orifice. *Arrowheads:* The ulcer scar at the anterior aspect of the duodenum bulb.

On day 13 of admission, an upper gastrointestinal contrast examination showed no obvious communication between the hepatic cyst and the duodenum (Fig. 2a). On day 20, CT showed that the hepatic cyst had tended to shrink but had not disappeared; in addition, the cyst wall had thickened (Fig. 2b). The CT also identified a low-density area between the hepatic cyst and the duodenum, which we suspected was a fistulous tract between them (Fig. 2c). On day 21, an upper gastrointestinal endoscopy revealed an ulcer scar at the anterior aspect of the duodenum bulb (Fig. 2d). A biopsy of the ulcer scar tissue revealed no malignancy.

**Figure 3:**
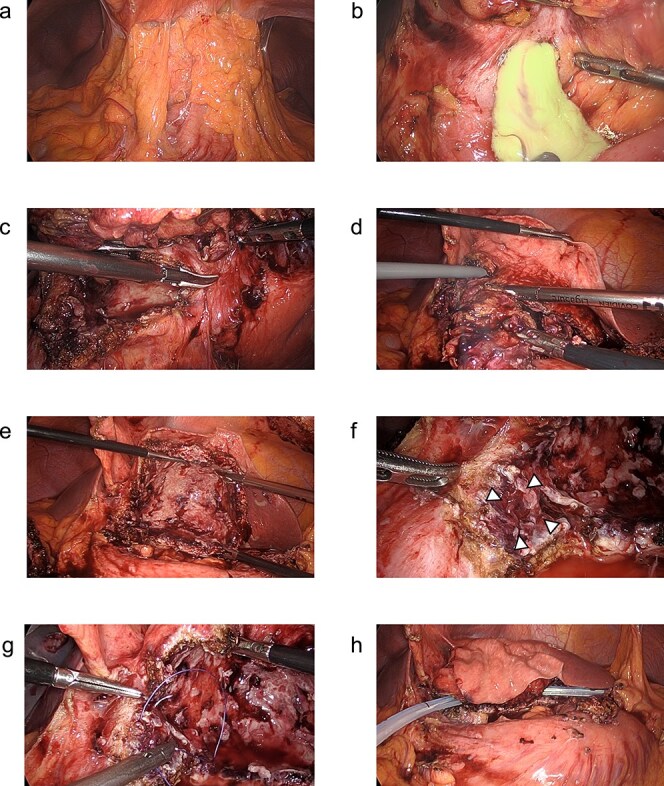
(a–h) Operative findings during the patient’s laparoscopic surgery. (a) Inflammatory tissue adhesions were observed on the peritoneum, omentum, and hepatic cyst. (b) A large amount of purulent fluid was discharged from the opened hepatic cyst. (c, d) The hepatic cyst wall was resected at the junction of the hepatic cyst and the liver parenchyma using the LigaSure™ vessel sealing system (Medtronic, Dublin, Ireland). (e) The laparoscopic hepatic cyst deroofing was completed. (f) A depression in the lumen of the fenestrated cyst near the duodenal bulb was observed, which was thought to be the penetration site between the hepatic cyst and the duodenum. *Arrowheads:* The depression in the lumen of the fenestrated cyst near the duodenal bulb. (g) The depression was closed using a 3–0 Vicryl® suture (Ethicon, Cornelia, GA, USA). (h) After the peritoneal lavage, one closed drain was placed at the site of the fenestrated hepatic cyst.

Because the hepatic cyst showed chronic inflammation and did not disappear, on day 42, the surgery was performed laparoscopically for the infected hepatic cyst (Fig. 3a). The cyst wall was opened, and a large amount of purulent fluid was discharged (Fig. 3b). Hepatic-cyst deroofing was performed (Fig. 3c–e). A depression was found in the lumen of the fenestrated cyst near the duodenal bulb, which was thought to be the penetration site between the hepatic cyst and the duodenum (Fig. 3f), and the depression was closed (Fig. 3g). One closed drain was placed at the site of the fenestrated hepatic cyst (Fig. 3h). The histopathological examination revealed no evidence of malignancy of the cyst wall.

**Figure 4:**
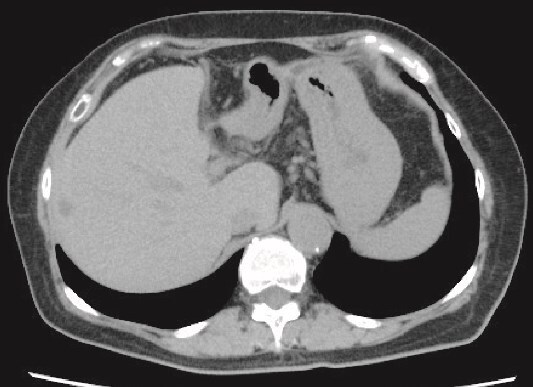
Plain CT image 6 months after the patient’s surgery. There was no evidence of hepatic cyst recurrence.

She was discharged home uneventfully on the 10th day after the surgery. CT has identified no evidence of recurrence 6 months postsurgery (Fig. 4).

## Discussion

We have described a rare case of a non-parasitic hepatic cyst infection involving the formation of a fistula between the hepatic cyst and a duodenum ulcer, treated with laparoscopic cyst deroofing. The patient’s postoperative course was uneventful, with no cyst recurrence within the 6 months after the surgery.

We considered the bacterial entry route of the hepatic cyst infection in the present patient’s case. An upper gastrointestinal contrast examination as well as an upper gastrointestinal endoscopy did not detect an obvious communication between the hepatic cyst and the duodenum, but CT revealed a low-density area between the hepatic cyst and the duodenum, which was suspected to be a fistulous tract between them. We concluded that (i) internal communication existed between the pre-existing hepatic cyst and the duodenal ulcer through a fistulous tract, and (ii) bacteria from the duodenum entered the hepatic cyst, which carried the cyst infection. Regarding the mechanism of fistula formation, we speculate that a spontaneous development of a duodenal ulcer might have penetrated the hepatic cyst; alternatively, the persistent compression and subsequent mechanical friction due to the large cyst might have resulted in a progressive erosion of the adjacent duodenal wall. As the patient’s duodenal ulcer healed, the obvious fistula between the hepatic cyst and the duodenal ulcer disappeared, with only an ulcer scar remaining.

There is a case report of a hepatic cyst infection with perforation of a duodenal ulcer into a non-parasitic liver cyst; the patient recovered completely with only conservative antibiotic treatment [4]. However, the use of only antibiotic treatment failed in many cases, with the patients eventually receiving further treatment [5]. A systematic review regarding the management of hepatic cyst infection [5] showed that most of the patients received antibiotic treatment initially, with a high rate of treatment failure, and ultimately, percutaneous cyst drainage or surgery was required [5]. In our patient, although antibiotic treatment and percutaneous cyst drainage were performed first, the chronic inflammation of the hepatic cyst infection persisted, and surgery was then conducted.

In this case, we performed infected hepatic cyst drainage percutaneously. However, with advancements in ultrasound endoscopy, transgastric drainage has been increasingly applied not only to biliary and postoperative pancreatic fistula drainage [6] but also to liver abscess drainage [7, 8]. If we experience a similar hepatic cyst infection as in this case in the future, we should consider a transgastric drainage approach.

Regarding surgical treatments for hepatic cyst infections, the above-cited review described both open and laparoscopic cyst deroofing and partial hepatic resection [5]. In our patient’s case, the duodenal ulcer had no malignancy. Considering the high risk of complications posed by a liver resection for the benign disease, we performed laparoscopic hepatic cyst deroofing.

Laparoscopic deroofing has become more commonly used for symptomatic hepatic cysts, and the anatomical suitability for laparoscopic deroofing should be considered. Major potential complications of laparoscopic surgery are hemorrhage and bile leakage [9]. Deeply situated cysts may communicate through a thin parenchyma wall with superficial cysts, and these cysts are both difficult to reach and difficult to differentiate from the vascular structure; they are not suitable for laparoscopic deroofing [2, 10, 11]. In addition, inadequate deroofing may encourage the recurrence of the hepatic cyst. Open deroofing is prudent for cystic lesions that are anatomically difficult to approach laparoscopically [2]. We performed laparoscopic deroofing in the present patient because her hepatic cyst was located in the lateral segments 2–3, which was considered to be applicable for a laparoscopic approach.

In conclusion, we have provided a successfully treated rare case of a hepatic cyst infection due to fistulous communication between a duodenal ulcer and a non-parasitic hepatic cyst. For patients with non-parasitic hepatic cyst infection, physicians should consider not only conservative antibiotic treatment but also surgical treatment including laparoscopic cyst deroofing.

## Supplementary Material

Supplementary_figure_1_rjaf484

Supplementary_figure_2_rjaf484
