# Supplementary material for: Laparoscopic deroofing to treat an infected hepatic cyst because of fistula formation between the hepatic cyst and the duodenum ulcer
Source: J Surg Case Rep. 2025 Jul 10;2025(7):rjaf484. doi: 10.1093/jscr/rjaf484 (PMC12240730; doi:10.1093/jscr/rjaf484)
Supplement: Supplementary_figure_1_rjaf484 [file supplementary_figure_1_rjaf484.pptx]

## Slide 1
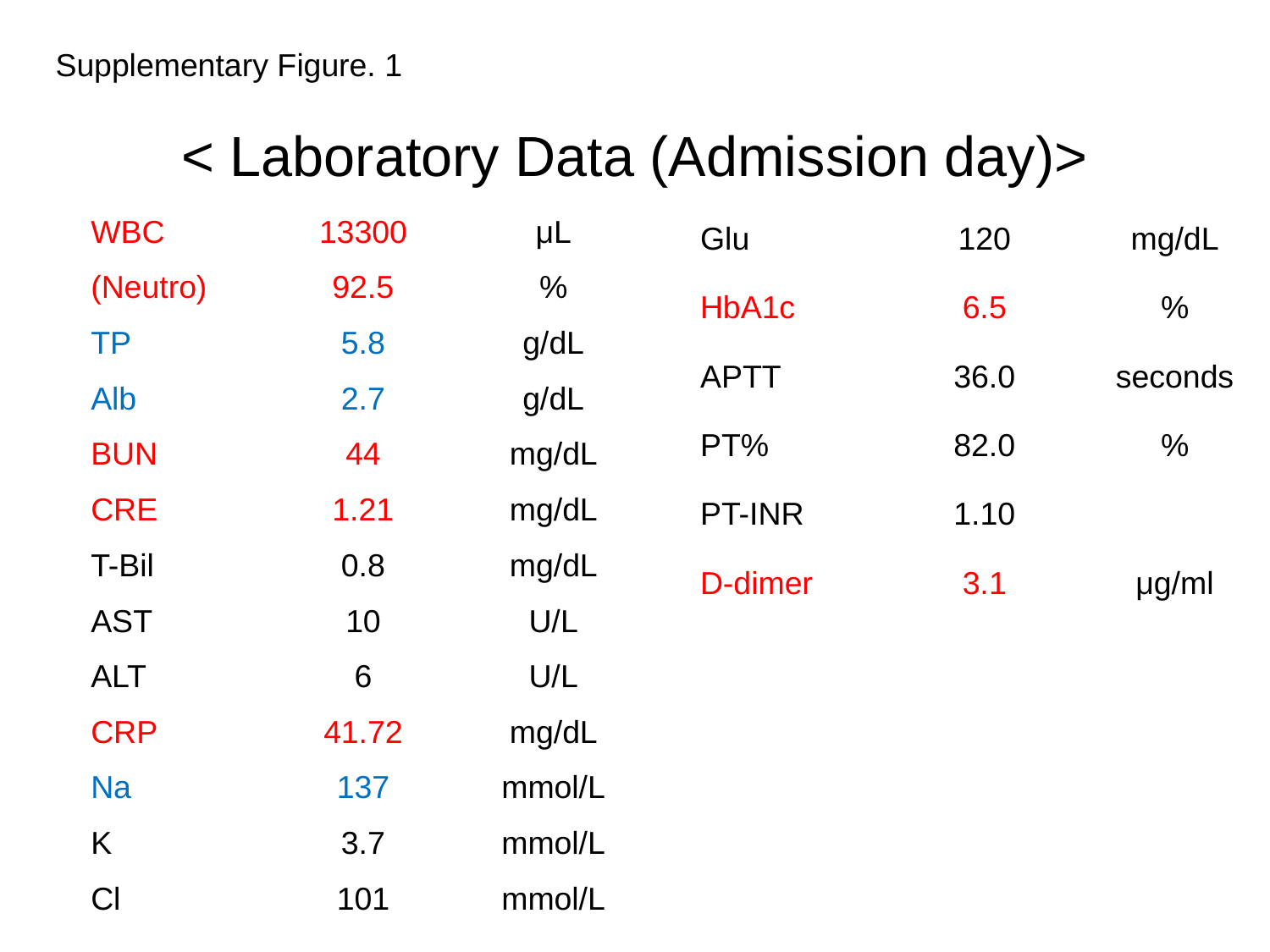

Supplementary Figure. 1
# < Laboratory Data (Admission day)>
| WBC | 13300 | μL |
| --- | --- | --- |
| (Neutro) | 92.5 | % |
| TP | 5.8 | g/dL |
| Alb | 2.7 | g/dL |
| BUN | 44 | mg/dL |
| CRE | 1.21 | mg/dL |
| T-Bil | 0.8 | mg/dL |
| AST | 10 | U/L |
| ALT | 6 | U/L |
| CRP | 41.72 | mg/dL |
| Na | 137 | mmol/L |
| K | 3.7 | mmol/L |
| Cl | 101 | mmol/L |
| Glu | 120 | mg/dL |
| --- | --- | --- |
| HbA1c | 6.5 | % |
| APTT | 36.0 | seconds |
| --- | --- | --- |
| PT% | 82.0 | % |
| PT-INR | 1.10 | |
| D-dimer | 3.1 | μg/ml |
