# Supplementary material for: Laparoscopic deroofing to treat an infected hepatic cyst because of fistula formation between the hepatic cyst and the duodenum ulcer
Source: J Surg Case Rep. 2025 Jul 10;2025(7):rjaf484. doi: 10.1093/jscr/rjaf484 (PMC12240730; doi:10.1093/jscr/rjaf484)
Supplement: Supplementary_figure_2_rjaf484 [file supplementary_figure_2_rjaf484.pptx]

## Slide 1
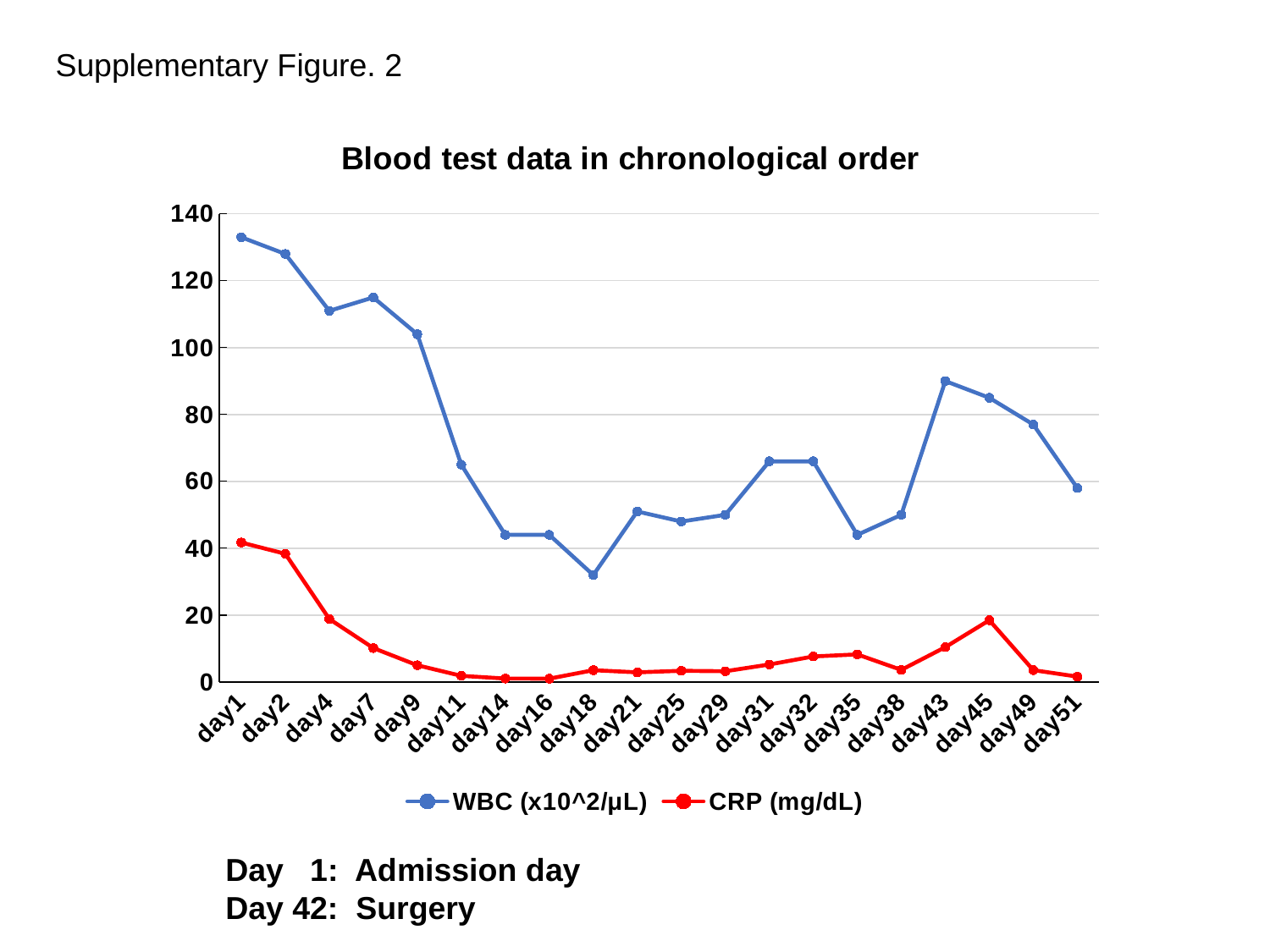

Supplementary Figure. 2
### Chart: Blood test data in chronological order
| Category | WBC (x10^2/μL) | CRP (mg/dL) |
|---|---|---|
| day1 | 133.0 | 41.72 |
| day2 | 128.0 | 38.35 |
| day4 | 111.0 | 18.86 |
| day7 | 115.0 | 10.16 |
| day9 | 104.0 | 5.03 |
| day11 | 65.0 | 1.86 |
| day14 | 44.0 | 1.05 |
| day16 | 44.0 | 1.02 |
| day18 | 32.0 | 3.55 |
| day21 | 51.0 | 2.9 |
| day25 | 48.0 | 3.37 |
| day29 | 50.0 | 3.23 |
| day31 | 66.0 | 5.25 |
| day32 | 66.0 | 7.66 |
| day35 | 44.0 | 8.27 |
| day38 | 50.0 | 3.64 |
| day43 | 90.0 | 10.43 |
| day45 | 85.0 | 18.49 |
| day49 | 77.0 | 3.56 |
| day51 | 58.0 | 1.63 |Day 1: Admission day
Day 42: Surgery
